# Brain size in birds is related to traffic accidents

**DOI:** 10.1098/rsos.161040

**Published:** 2017-03-29

**Authors:** Anders Pape Møller, Johannes Erritzøe

**Affiliations:** 1 Ecologie Systématique Evolution, Université Paris-Sud, CNRS, AgroParisTech, Université Paris-Saclay, 91405 Orsay Cedex, France; 2 House of Bird Research, Taps, Christiansfeld 6070, Denmark

**Keywords:** birds, brain mass, traffic, viability selection

## Abstract

Estimates suggest that perhaps a quarter of a billion birds are killed by traffic annually across the world. This is surprising because birds have been shown to learn speed limits. Birds have also been shown to adapt to the direction of traffic and lane use, and this apparently results in reduced risks of fatal traffic accidents. Such behavioural differences suggest that individual birds that are not killed in traffic should have larger brains for their body size. We analysed the link between being killed by traffic and relative brain mass in 3521 birds belonging to 251 species brought to a taxidermist. Birds that were killed in traffic indeed had relatively smaller brains, while there was no similar difference for liver mass, heart mass or lung mass. These findings suggest that birds learn the behaviour of car drivers, and that they use their brains to adjust behaviour in an attempt to avoid mortality caused by rapidly and predictably moving objects.

## 1. Introduction

An extensive review of the literature on road casualties revealed effects of location, survey method, year and season on the number of traffic casualties [1]. Abundance was the single most important predictor of the frequency of traffic casualties in birds [2]. Once this variable had been accounted for, species with short flight initiation distances, estimated as the distance at which birds took flight when approached by a human, solitary species, species with high prevalence of malarial parasites, duration of the nestling period and species with a relatively large bursa of Fabricius accounting for the B-cell immunity repertoire in birds explained additional variation in frequency of road kills [2].

Traffic has only been of any significance for survival of animals during the last century, and this has caused some scientists to claim that this cause of mortality cannot possibly have changed as a result of selection. Some studies have shown that risk-taking behaviour and sociality may account for residual variation in the risk of traffic casualties [2]. This implies that there may have been response to selection linked to micro-evolutionary changes in escape behaviour [3] and hence the risk of traffic accidents. Given that perhaps 365 million animals die annually from traffic accidents in the USA alone [4,5], and more than 57 million birds are killed annually in Europe [5] (table 1), these estimates may suggest intense selection. The risk of mortality from traffic may partly be due to cognitive abilities because individuals belonging to species with relatively large brains for their body size may have better abilities to avoid complex means of encounters such as the approach by hunters and their use of guns to kill birds [6]. Rapidly approaching predators, cars and other approaching objects may likewise favour individual animals that have superior cognitive abilities.

**Table 1.** Probability of birds being killed by traffic in relation to residual brain mass (covariate), age and sex (fixed factors) and species (random factor) across all species of birds. Residual brain mass was residuals from a regression of log-transformed brain mass on log-transformed body mass. Sample size was 3524 with an adjusted *R*^2^ of 0.29. The variance component for species was 0.049, with s.e. = 0.008, 95% CI 0.034–0.064, accounting for 24% of the variance.

term	*F*	d.f.	*p*	estimate	s.e.
intercept		207.1	<0.0001	0.205	0.020
residual brain mass	19.24	1090	<0.0001	−0.353	0.081
age (adult)	3.92	3513	0.048	0.015	0.007
sex (female)	0.01	3493	0.933	−0.001	0.007

In contrast to selection, there is evidence of phenotypic adjustment to traffic. For example, birds have learnt to adjust their risk-taking behaviour to speed limits on roads [7]. In addition, carrion-feeding crows fly up when located in a lane with fast-moving cars, but stay put in the opposite lane. Crows in the same lane as cars often walk to the opposite lane. Therefore, crows can detect directionality of an oncoming car implying that crows understand the behaviour of vehicular traffic [8]. These observations are consistent with behavioural adjustment to traffic in free-living animals.

Here we assessed the intensity of selection on brain size due to traffic accidents, and in a second analysis we repeated the analysis after excluding birds that had been shot by hunters [6]. In addition, we provided a first estimate of the global rate of mortality in any class of animals caused by traffic.

## 2. Material and methods

### 2.1. Specimens used for this study

J.E. recorded the site, the date when a specimen was received and the cause of death as reported by the person delivering the bird (J.E. verified by a subsequent autopsy the cause of death as reported by the person delivering the specimen, or if the bird was delivered without any cause of death reported, this was subsequently determined by J.E. in an autopsy). All specimens were analysed by a single observer, hence eliminating any among-observer variance. Most specimens originated from 1960 to 2015 from an area surrounding Christiansfeld, Denmark. The wet body mass of each specimen was weighed to the nearest 0.1 g, while the extracted brain was weighed to the nearest 0.01 g. None of the specimens included in this study had a damaged head from hunting or other causes of death. Further information on the procedures has been reported previously [9]).

Age was scored as juvenile or adult according to standard criteria reported by Svensson [10]. All data on brain size were collected without prior knowledge of the cause of death, and hence there was no possibility of conscious or unconscious bias. The data are reported in the Dryad Digital Repository [11].

### 2.2. Statistical analyses

We used generalized linear mixed models (GLMMs) with cause of death due to traffic (0—no, 1—yes) as a dichotomous response variable that is binomially distributed, while species was entered as a factor to account for large differences in sampling effort among species, age and sex were used as fixed factors, and residual brain mass and body mass were used as continuous covariates. Because brain mass and body mass were strongly positively correlated with a variance inflation factor (VIF) of 8.39 for body mass and 8.45 for brain mass, we used residual brain mass from a model that included log-transformed brain mass as a response variable, and species as a random effect. This analysis had a maximum VIF at 1.03, thus providing little evidence of collinearity. Finally, we performed an analysis with traffic-killed or not as the response variable after exclusion of shot birds, sex and age as fixed factors, residual brain mass as a continuous covariate and species identity as a random effect. All analyses were made with JMP [12].

## 3. Results

**Figure 1.**
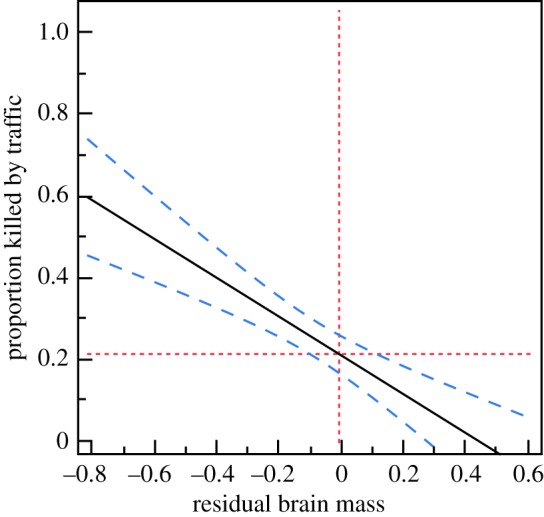
Prediction profiler showing the probability of birds getting killed by traffic in relation to relative log-transformed brain mass after controlling statistically for the random effect of species, the fixed effect of age and sex, and the effect of the covariate body mass. The line is the regression line; the 95% CIs for the predicted relationship are shown by blue hatched lines, and the red lines are the mean values for the predictor and the response variables.

**Figure 2.**
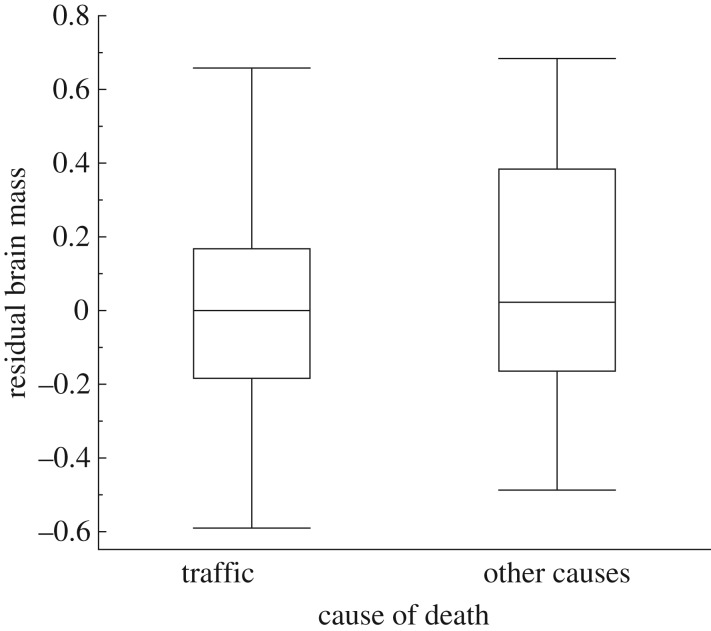
Box plots of residual brain mass for birds that were killed in traffic or died from other causes. Residual brain mass was the brain mass after controlling statistically for the random effect of species, the fixed effect of age and sex, and the effect of the covariate body mass. Box plots show the median, quartiles, 5- and 95-percentiles and extreme data points.

Residual brain size strongly predicted collision mortality in birds, with a negative relationship between residual brain size and proportion of individuals killed (adjusted *r*^2^ = 0.29; figure 1). The proportion of individual birds killed by traffic decreased from 60% to 0% across the range of relative brain sizes recorded (figure 1). Box plots of residual brain size (adjusted for the variables in table 1) for birds that had been killed by traffic or those that died from other causes showed a larger top quartile for individual birds that had been killed by traffic than for those that died from other causes (figure 2). In contrast, there was no significant link between risk of collision with vehicles and sex, while there was a marginally significant effect of age (table 1). The risk of being killed by a hunter has been shown to be related to relative brain size [6]. The probability of being killed in traffic might not be independent of the probability of being killed by a hunter. Therefore, we excluded all specimens killed by a hunter. The conclusions remained the same in this restricted dataset (electronic supplementary material, table S1). The effects of sex and age did not reach statistical significance (electronic supplementary material, table S1).

In contrast to the effect of brain mass, there was no similar effect for liver mass (*F* = 0.47, d.f. = 1, 2339, *p* = 0.49), heart mass (*F* = 0.88, d.f. = 1, 1563, *p* = 0.35) or lung mass (*F* = 0.53, d.f. = 1, 140.6, *p* = 0.47).

## 4. Discussion

Birds killed by traffic had consistently smaller brains than survivors. This difference was specific to brain size, but did not apply to the size of liver, heart or lungs. The difference in brain size was between individuals killed by traffic and other individuals [13]. This observation is consistent with a difference in brain size between individuals killed from being shot or from traffic, both being due to cognitive differences.

However, such differences in brain mass due to the risk of death caused by traffic or the risk of being shot do not imply that such mortality may result in micro-evolutionary change. Indeed, mortality due to traffic most likely only accounts for a small fraction of overall mortality [14]. We can make the following estimates. How many birds are there in the world? Estimates suggest that 57 million are killed in Europe alone [1] (table 1). If similar numbers are killed in each of the continents North America, South America, Asia, Africa and Australia by road kills, this gives a total of 342 million. Then the mortality rate due to traffic is given by 342 million/300 000 million or 0.114% if we assume that there are 300 000 million birds in the world [15]. This is an exceedingly small mortality that is unlikely to have a significant impact on micro-evolution even if we consider the fact that such mortality is not distributed evenly in space and time.

A cognitive study of behaviour in birds in relation to the risk of death caused by traffic has shown adjustment in risk-taking behaviour of individual birds to speed limits [7]; also crows adjust their choice of lane use according to that used by drivers and to the specific behaviour of drivers [8]. Such behavioural adjustment of individual birds to the specifics of traffic is likely to have an underlying neural substrate. Here we have shown that there is a link between being killed in traffic accidents and the relative size of the brain. This may suggest that there is a link between behaviour, relative brain size and viability.

In conclusion, birds killed by cars have disproportionately small brains for their body size. In contrast, there was no difference in terms of the size of the liver, heart or lungs. Both road kills and birds being shot had disproportionately small brains for their body size. These findings suggest that cognitive differences between dead individuals and survivors may be linked to individual differences in perception and adjustment to movement.

## Supplementary Material

ESM Table 1

## Data accessibility

Data are available from the Dryad Digital Repository, http://dx.doi.org/10.5061/dryad.j075c [11].
