# Supplementary material for: Brain size in birds is related to traffic accidents
Source: R Soc Open Sci. 2017 Mar 29;4(3):161040. doi: 10.1098/rsos.161040 (PMC5383851; doi:10.1098/rsos.161040)
Supplement: ESM Table 1 [file rsos161040supp1.docx]

**ESM Table 1.** Probability of birds being killed by traffic in relation to residual brain mass (covariate), age, sex and species (random factor) across all species of birds after exclusion of specimens that were shot. Residual brain mass was residuals from a regression of log-transformed brain mass on log-transformed body mass. Sample size was 3220 with an adjusted *R*^2^ of 0.29. The variance component for species was 0.054, SE = 0.009, 95% CI 0.037 to 0.071, accounting for 25% of the variance.

| Term | *F* | df | *P* | Estimate | SE |
| --- | --- | --- | --- | --- | --- |
| Intercept |  | 169.6 | < 0.0001 | 0.266 | 0.023 |
| Residual brain mass | 44.20 | 1313 | < 0.0001 | -0.609 | 0.091 |
| Age [Adult] | 3.18 | 3200 | 0.075 | 0.014 | 0.008 |
| Sex [Female] | 0.66 | 3191 | 0.420 | -0.006 | 0.008 |
